# Layer thickness prediction and tissue classification in two-layered tissue structures using diffuse reflectance spectroscopy

**DOI:** 10.1038/s41598-022-05751-5

**Published:** 2022-02-01

**Authors:** Freija Geldof, Behdad Dashtbozorg, Benno H. W. Hendriks, Henricus J. C. M. Sterenborg, Theo J. M. Ruers

**Affiliations:** 1 Department of Surgery, Netherlands Cancer Institute, 1066 CX Amsterdam, The Netherlands; 2 Department of IGT and US Devices & Systems, Philips Research Laboratories, 5656 AE Eindhoven, The Netherlands; 3 Department of BioMechanical Engineering, 3mE, Delft University of Technology, 2628 CD Delft, The Netherlands; 4 Department of Biomedical Engineering and Physics, Amsterdam University Medical Center, 1105 AZ Amsterdam, The Netherlands; 5 Faculty of Science and Technology, University of Twente, 7522 NB Enschede, The Netherlands

**Keywords:** Surgical oncology, Near-infrared spectroscopy, Imaging and sensing

## Abstract

During oncological surgery, it can be challenging to identify the tumor and establish adequate resection margins. This study proposes a new two-layer approach in which diffuse reflectance spectroscopy (DRS) is used to predict the top layer thickness and classify the layers in two-layered phantom and animal tissue. Using wavelet-based and peak-based DRS spectral features, the proposed method could predict the top layer thickness with an accuracy of up to 0.35 mm. In addition, the tissue types of the first and second layers were classified with an accuracy of 0.95 and 0.99. Distinguishing multiple tissue layers during spectral analyses results in a better understanding of more complex tissue structures encountered in surgical practice.

## Introduction

In oncological surgery, the aim is usually to remove the entire tumor together with a margin of healthy tissue, while other surrounding healthy structures are spared as much as possible. However, identifying the tumor during surgery can be challenging. Inadequate tumor resection increases the risk of local tumor recurrence and decreases survival rates, making the resection margin status an important prognostic factor for patient outcome^1^. This emphasizes the demand for a real-time tissue discrimination technique to provide intra-operative guidance.

In the last decades, optical techniques have been introduced to analyze the composition of materials in various fields, such as environmental monitoring by remote sensing^2–4^, agriculture^5,6^, food quality inspection^7–9^, chemical and pharmaceutical industry^10,11^, and forensics^12,13^. In the medical field, multiple optical techniques have been introduced for tissue discrimination and margin assessment. Examples include hyperspectral imaging^14,15^, elastic scattering spectroscopy^16,17^, and Raman spectroscopy^18,19^. Common advantages of these optical techniques are that they are fast, non-invasive, and do not require administration of contrast agents. Another such technique is diffuse reflectance spectroscopy (DRS)^20–36^. DRS is a point-based technique, in which broadband light with wavelengths in the visible and/or near-infrared range is sent into tissue using optical fibers. After the light undergoes various scattering and absorption events in the tissue, a part of the light is reflected back to the tissue surface. The detected diffuse reflectance spectrum of this light represents an “optical fingerprint” of the measured tissue, which can be analyzed. DRS has been successfully evaluated for detection of cancer in breast^20,21,29^, colorectal^30–34^, head and neck^22,35,36^, liver^23,24^, lung^25,26^, and brain^27,28^ tissue, in both ex vivo and in vivo studies. These studies showed that tumor tissue could be discriminated from healthy tissue with classification accuracies of 0.77–1.00, suggesting this technique has a great potential for real-time tissue assessment during surgery.

The aforementioned studies focused primarily on identifying the tissue directly at the measurement surface, assuming a single-layer homogeneous tissue structure. When analyzing the DRS spectra, often fit models were used to estimate optical and physiological parameters of the measured tissue such as water, fat, hemoglobin, β-carotene and collagen concentration, reduced scattering amplitude, and mie scatter slope. In surgical practice, however, the measured tissue is often inhomogeneous and may consist of several layers, which decreases the performance of current analytical models, such as described by Farrell et al.^37,38^.

In previous studies, different DRS models for two-layered media have been proposed, mainly to analyze multi-layered skin epithelium^39–47^. However, most of these studies are based on only Monte Carlo simulations to generate diffuse reflectance spectra of two-layered models with various physiological parameters and did not use any experimental data as input. Inverse fitting was then used to quantify and validate the optical properties and epidermal thickness, some combined with liquid phantom measurements^40,41^ or comparisons with known values for human skin^44–46^. The large number of parameters in these two-layer models greatly increases the computational time, which is not desirable for real-time in vivo use during surgery. Moreover, the modeled epidermal layer thicknesses were in the order of 100 µm, while during surgery, layer thicknesses of multiple millimeters can be encountered.

In recent years, machine learning models are progressively replacing physical models. This paper will investigate whether advances in machine learning can address the challenges in analyzing two-layered tissue structures, using experimental data from both a two-layered phantom and two-layered animal tissue. Distinguishing multiple tissue layers during spectral analyses would ultimately facilitate the analysis of more complex tissue structures and the assessment of complete resection margins up to multiple millimeters in depth. First, the feasibility of extracting structural information from fiberoptic DRS data will be examined. To this end, we will explore whether DRS can be used to predict the thickness of the top layer in two-layered tissue. This will be performed in a controlled experiment, using a two-layered tissue-mimicking phantom. To the best of our knowledge, no similar approaches have been explored before. In clinical practice, the tissue type discrimination of the different layers is important as well. Therefore, a second experiment will be performed using animal tissue, containing a layer of fat and a layer of muscle tissue. In addition to predicting the top layer thickness, we will examine whether it is possible to predict the tissue types of both the top layer and the layer beneath it. Lastly, the thickness prediction performance using DRS will be compared to the performance using ultrasound (US) imaging, and to a combination of both techniques.

This study aims to use DRS to predict the top layer thickness in two-layered tissue structures and classify the tissue type of both layers. After acquiring the data, two types of features will be extracted and used in regression and classification models to evaluate the performances of the developed machine learning algorithms.

## Methods

### Measurement setup

The fiberoptic DRS system consisted of a Tungsten halogen broadband light source and two spectrometers; one for the visible domain (400–1100 nm, Andor Technology, DU420ABRDD) and one for the infrared domain (900–1700 nm, Andor Technology, DU492A-1.7)^48^. A probe with two source-detector fiber distances of 2 and 6 mm was used to retrieve information from both superficial and deeper sampling depths. In DRS, the sampling depth (penetration depth of the detected photons) depends on the distance between the source and detector fiber. Even though the sampling depth is also influenced by the sample optical properties, it has been shown that the sampling depth is approximately equal to the source-detector distance^49^. Ultrasound images were acquired using the portable Philips CX50 machine (Philips Research, Eindhoven, The Netherlands) in combination with the Philips L15-7io transducer (Philips Research, Eindhoven, The Netherlands), a high-frequency 15–7 MHz ultrasound transducer specially designed for superficial imaging.

### Phantoms

#### Tissue-mimicking phantom

**Figure 1:**
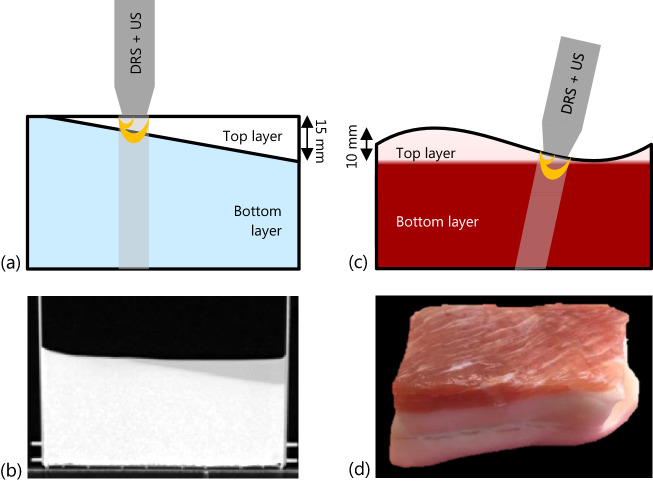
(**a**) Schematic representation of an artificial phantom, consisting of two different homogeneous layers with a gradually increasing top layer thickness; (**b**) axial view of CT-scan of the artificial phantom; (**c**) schematic representation of two-layered animal tissue, with a varying top layer thickness; (**d**) picture of a piece of animal tissue, with a top layer of muscle tissue and a bottom layer of fat tissue.

For the first phase of this study, an artificial phantom was created that consisted of two homogeneous layers with a sharp boundary in between. The thickness of the top layer gradually increased from 0 to 15 mm, see Fig. 1a. For both layers, 70% polyacrylamide gel (100 mg/mL water) was used as base material and 30% Intralipid (20% stock solution) was added for fat contrast. In the bottom layer, patent blue (5 µg/mL) and BaSO4 particles (20 mg/mL) were added for optical contrast in the visible part of the spectrum and US/X-ray contrast, respectively. A CT scan of the artificial phantom was made as ground truth for dimensions (Fig. 1b), using a clinical CT scanner (Siemens Sensation Open, Siemens Medical Solutions, Erlangen, Germany).

#### Animal tissue

For the second phase of this study, multiple pieces of two-layered bovine and porcine tissue were used. The tissue consisted of a thick bottom layer and a thin top layer with a thickness varying between 0 and 10 mm. Two types of animal tissue phantoms were examined; muscle tissue with a top layer of fat and fat tissue with a top layer of muscle, as demonstrated in Fig. 1c,d. The animal tissue was obtained from a local supermarket, so no ethical approval was required.

### Data acquisition and preprocessing

DRS and US measurements were performed at fifteen locations on the artificial phantom. Data was acquired using four different probe orientations per measurement location, each time the probe was turned a quarter turn. A grid with cutouts of the desired probe locations and orientations was used to ensure that the center of the probes remained at the same location when changing orientations. All measurements were repeated three times, which resulted in 180 measurements in total. The ground truth for the top layer thickness was determined based on the obtained CT scan. The corresponding axial CT slide was extracted for each measurement location, and the top layer thickness was measured using annotation tools and then converted from pixels to millimeters based on CT image resolution.

In the second phase, measurements were performed in a grid pattern at multiple locations on the animal tissue. First, fiberoptic DRS measurements were performed at every location. Since it was not possible to obtain CT images for animal tissue samples, the ground truth for the top layer thickness was determined by US imaging. To this end, an US image was acquired in such a way that the center of the US image corresponded to the location of the DRS measurement. DRS and US measurements were performed at 250 locations in total; 122 fat on top of muscle measurements and 128 muscle on top of fat measurements. The top layer thickness was manually measured in the US image. All measurement locations associated with a top layer thickness smaller than 6 mm (n = 186) were included for further analyses since it is expected that the sampling depth of DRS will be roughly equal to the maximum fiber distance of 6 mm^49^ and there is also no clinical interest to assess tissue deeper than 6 mm. The average top layer thicknesses were equal to 2.81 ± 1.90 mm (0–5.95 mm) and 3.25 ± 1.57 mm (0–5.96 mm) for top layers of fat and muscle, respectively.

After data acquisition, the DRS spectra from the visible and near-infrared wavelength ranges were stitched together to create one spectrum for each measurement location, ranging from 400 to 1600 nm (1200 features). The data from 1600 to 1700 nm was removed, due to a low signal-to-noise ratio. The spectra were calibrated using white and dark reference measurements, which were acquired at the beginning of every measurement session, see Eq. (1):

1 $${R}_{cal}= \frac{{R}_{meas} - {R}_{dark}}{{R}_{white}- {R}_{dark}},$$

where ${R}_{cal}$ is the calibrated measurement, ${R}_{meas}$ is the uncalibrated measurement, ${R}_{dark}$ is the dark reference measurement and ${R}_{white}$ is the white reference measurement. The white reference measurement was obtained using Spectralon (Avantes WS-2, Avantes, Apeldoorn, The Netherlands), the dark reference measurement was obtained by switching off the fiberoptic light source. The spectra were normalized with respect to the reflectance value at 800 nm to compensate for any intensity differences that might have been present. This wavelength was chosen since we do not expect any significant absorption to be present in this region.

### Data analysis

In this subsection, we will introduce the methodology for extracting features from DRS spectra and US images. The extracted features will be used to train a regression analysis and tissue classification model for a two-layered structure. The feasibility of predicting layer thicknesses based on DRS spectra was evaluated first in a controlled setting using an artificial phantom. As a next step, two-layered animal tissue was used to facilitate tissue classification of the different layers as well.

**Figure 2:**
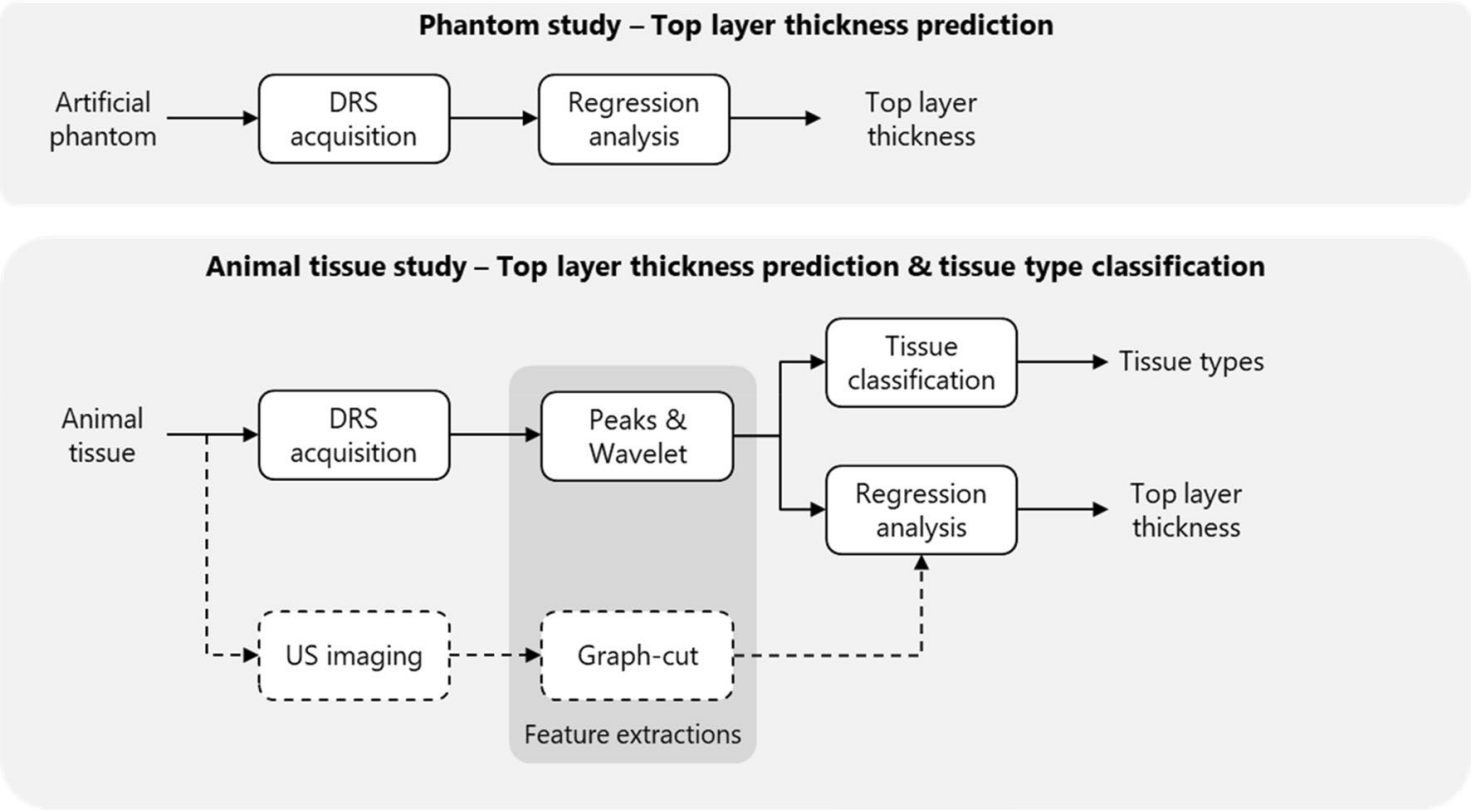
Workflow of data acquisition and data analysis for the top layer thickness estimation and tissue type classification. In the phantom study, DRS data was used as input for regression analysis, in which the top layer thickness was predicted. In the animal tissue study, two types of features were extracted from the DRS data. These features were used in a classification analysis to predict the tissue type of both the 1st and 2nd tissue layer and in a regression analysis to predict the top layer thickness. In addition, these results were compared to the results of using US imaging and using both techniques combined. Therefore, US images were acquired and structural features were extracted based on a graph-cut method.

Because of the less complex situation in the artificial phantom, no feature extraction methods have been performed in this phase and no US features were used. In the more complex phase with animal tissue, two different DRS feature extraction methods were applied and the obtained features from the small and large fiber distances were combined. The added value of US features was evaluated as well in the second phase of this study. The complete pipeline of data acquisition and data analysis is demonstrated in Fig. 2.

#### Spectral feature extraction

**Figure 3:**
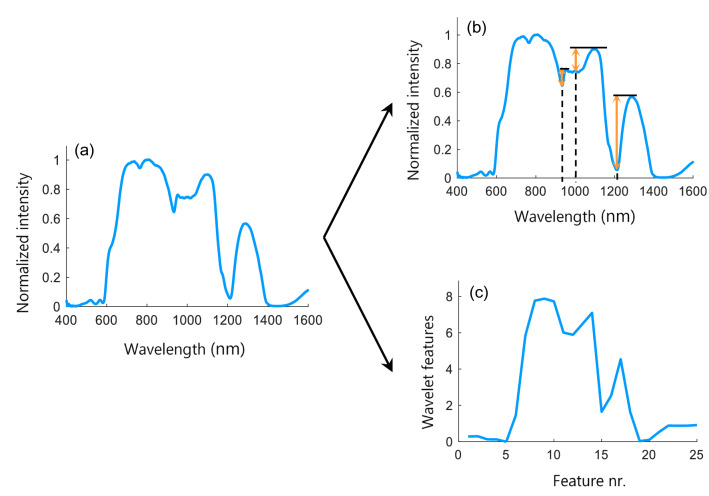
DRS feature extraction methods. (**a**) Original DRS spectrum, consisting of 1200 wavelength features; (**b**) the dip-peak heights for three spectral regions: 935 nm, 985 nm and 1200 nm, resulting in 3 features; (**c**) level 6 approximation spectrum, resulting in 25 features.

One DRS measurement consists of 1200 wavelength features as demonstrated in Fig. 3a. To effectively reduce the amount of data and to reduce the risk of overfitting, two different spectral feature extraction techniques were examined in this study; feature extraction based on spectral peaks and based on the wavelet transform. For the peak-based method, the largest dip to peak distance was calculated for three spectral regions with distinct peaks: 935 nm, 985 nm and 1200 nm as demonstrated in Fig. 3b. These wavelength regions were chosen for their dominant water and fat absorption in different ratios. The peak heights were calculated by subtracting the minimum intensity value from the maximum intensity value in the regions 920–960 nm, 960–1150 nm, and 1150–1325 nm, respectively. By concatenating the three peak heights for both fiber distances, this method resulted in six features.

The second feature extraction method was based on the wavelet transform, which can be used to analyze spectra at different spectral scales. For this study, the dyadic implementation of the transform, as described by Denstedt et al*.*, was used^50^. The input spectra were convolved with a Symlet low-pass filter of size 8 to obtain approximation spectra. Subsequently, the obtained spectra were down-sampled by a factor of 2 and the procedure was repeated multiple times, each time using the approximation spectrum of the previous iteration. The approximation spectra of the 6th iteration, consisting of 25 features, were used for the analyses (Fig. 3c). Extracting these features for both fiber distances resulted in 50 features in total.

#### Ultrasound feature extraction

**Figure 4:**
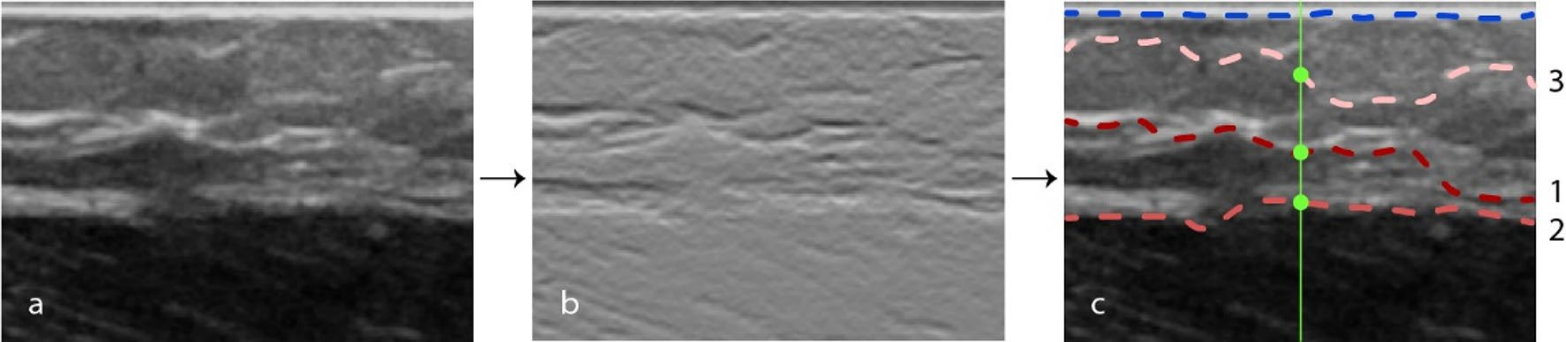
US feature extraction workflow. (**a**) Original US image of a fat layer on top of muscle measurement; (**b**) vertical gradient image; (**c**) result of applying the graph theory algorithm. The blue line represents the contact surface as selected by the graph theory, which is excluded from further analysis. The red lines represent the three best paths that were selected. The numbers indicate the selection order, with path 1 being the first path selected with the lowest cost. The green line represents the DRS measurement location in the center of the US image, with the feature extraction points.

To be able to determine the added value of US imaging for predicting the layer thickness, features were extracted from the US images based on the graph-cut theory. In this technique, an image is represented as a graph with nodes and edges representing the individual pixels and the connections between two neighboring pixels, respectively. The specific method used in this study was based on an automatic segmentation algorithm provided by Chiu et al*.*^51^. Although the algorithm was designed for the segmentation of retinal layers, it is suitable for layered structures in general. Some modifications were made in order to find the fat to muscle and muscle to fat boundaries. First, the image was smoothed using a Gaussian filter ($5x5; \sigma =1$) and then the vertical gradient image was obtained, see Fig. 4b. Subsequently, a weight was assigned to each of the graph edges to create path preferences, in such a way that the edges of pixel pairs with the highest vertical gradients have the lowest weights. Boundaries were selected based on minimal cost, i.e. the path with the highest vertical gradients, for which Dijkstra's algorithm was used^52^. The upper left and bottom right corner pixels are used as endpoints for the path selection. Therefore, two columns with minimal weights are padded left and right to the image to obtain an adequate graph cut, as explained by Chiu et al*.*^51^.

The path corresponding to the contact surface between the transducer and the tissue was selected using the first twenty pixel rows only (blue path in Fig. 4c). In the remaining part of the image, the tissue boundary was selected. Speckle noise and the presence of tissue fibers in the ultrasound images can sometimes result in the selection of other, incorrect, paths that do not represent a tissue transmission boundary. The best path, with the lowest cost, does therefore not always correspond to the correct path. To avoid missing the correct path, the three best paths were selected in each image. In this way, the correct path is always taken into account during the analysis. Figure 4c shows an example of the three selected paths. In this case, the second selected path is the correct boundary.

The three selected boundaries were translated into six features that were used to estimate the top layer thickness. For each of the three selected boundaries, the distance from the contact surface and the vertical gradient value were used as feature values. This resulted in the following six features: (1) distance from the first boundary to the contact surface, (2) distance from the second boundary to the contact surface, (3) distance from the third boundary to the contact surface, (4) vertical gradient value at the first boundary, (5) vertical gradient value at the second boundary, (6) vertical gradient value at the third boundary. These values were extracted in the center of the US image at the location of the paths, as demonstrated in green in Fig. 4c.

#### Top layer thickness prediction

The top layer thickness of the artificial phantom was predicted using a Gaussian process regression analysis. All 1200 DRS wavelengths were used as features. The regression was performed twice; for a fiber distance of 2 mm and a fiber distance of 6 mm. The average and standard deviation of the prediction error were used for the evaluation using an iterated 10-fold cross-validation technique, in which the results were averaged over 20 iterations. Per location, all spectra were assigned to one fold to ensure that spectra from one location were not split between the training and test set.

For the top layer thickness estimation of the animal tissue, the data set was divided into two groups. One group consisted of the muscle measurements with and without a top layer of fat and one group consisted of the fat measurements with and without a top layer of muscle. For each group separately, the thickness of the top layer was estimated using regression analysis and the combined features of both fiber distances. Again, an iterated cross-validation technique was used and the results were averaged over the 20 iterations. The performances of two regression models were compared; a linear support vector machine (SVM) model and a Gaussian process regression (GPR) model. The regression models were first trained using only DRS features and then using both DRS and US features to examine the added value of US imaging. A Wilcoxon signed-rank test was performed to examine the presence of any significant differences between the results of the two classification models or the different feature sets. The significance threshold was set at 0.05.

#### Tissue classification

The tissue classification was only performed on animal tissue. All 186 measurements on animal tissue were used for tissue classification of the first layer; 89 with a top layer of fat and 97 with a top layer of muscle. Tissue classification of the second layer was performed using only the two-layered measurements (73 with a second layer of fat and 81 with a second layer of muscle). The tissue types (muscle/fat) of both layers were classified independently of each other using quadratic SVM classification models, based on the DRS features only. An iterated 10-fold cross-validation was performed and the results were averaged over the 20 iterations. The classifier was trained once using the peak features and once using the extracted wavelet features. The classification performance was evaluated using the accuracy, Matthews correlation coefficient (MCC), the area under the curve (AUC), sensitivity, and specificity.

## Results

### Tissue-mimicking phantom

**Figure 5:**
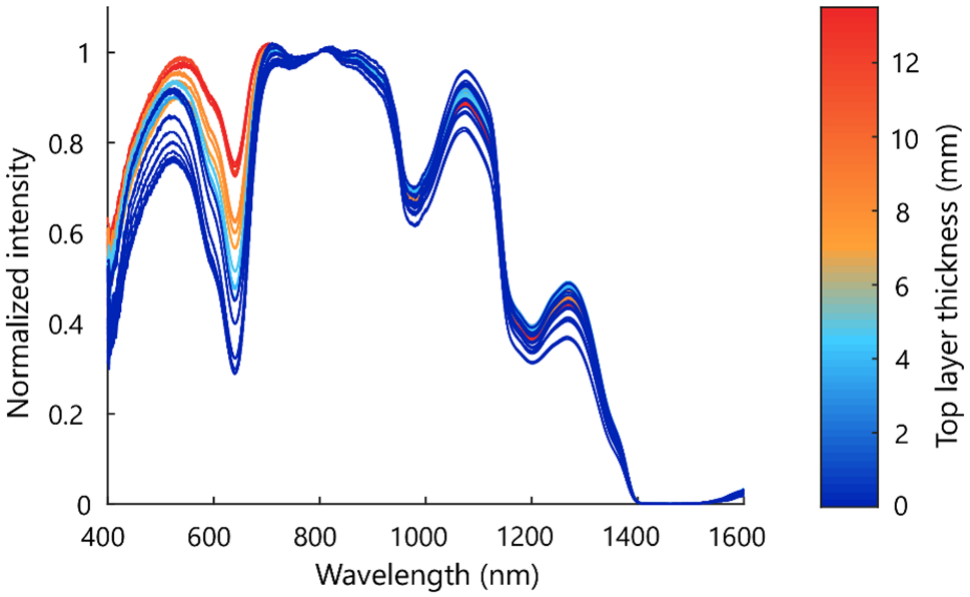
Normalized diffuse reflectance spectra of different locations on the artificial phantom, using a fiber distance of 2 mm. The color represents the thickness of the top layer at the measured location. The bottom layer contained patent blue, with a peak absorption at 635–640 nm.

DRS measurements were performed at fifteen locations on the artificial phantom, consisting of a top layer with gradually increasing thickness and a bottom layer with patent blue. The resulting DRS spectra are shown in Fig. 5. The color represents the top layer thickness at the measured location, which corresponds to how far the patent blue layer was located beneath the surface. Patent blue has a peak absorption at 635–640 nm. For all top layer thicknesses, a clear dip is visible in the measured reflectance spectrum at these wavelengths. In addition, differences can be seen in the size of the dip for the different top layer thicknesses. The thinner the top layer, the more absorption has occurred in this wavelength area.

**Figure 6:**
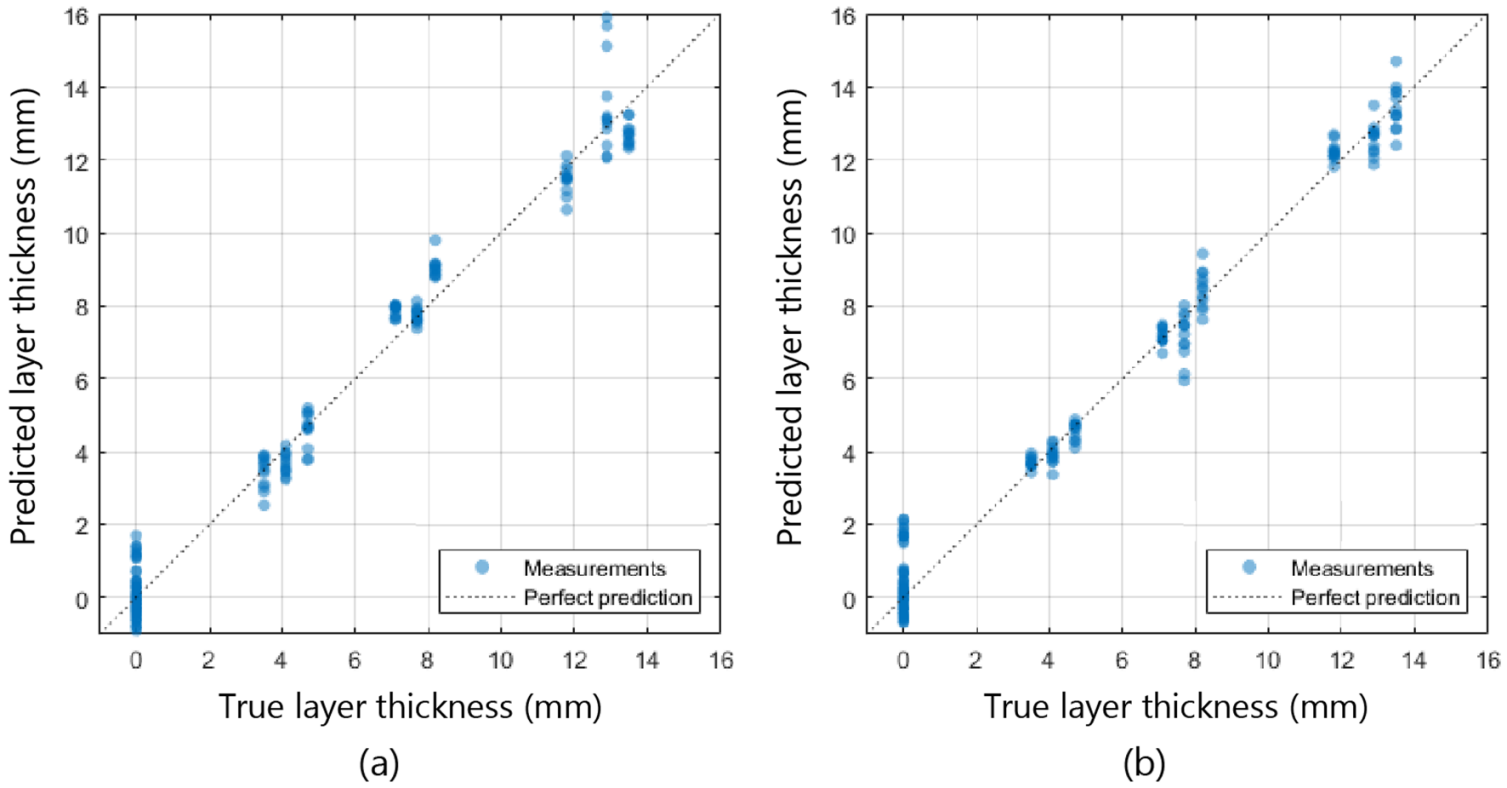
Predicted top layer thickness compared to the true top layer thickness, for a fiber distance of 2 mm (**a**) and 6 mm (**b**).

Using the regression analysis as described in “Data analysis”, the top layer thickness could be estimated with a mean prediction error of 0.71 ± 0.07 mm for a fiber distance of 2 mm and 0.69 ± 0.04 mm for a fiber distance of 6 mm. The predicted top layer thickness is plotted against the true top layer thickness for all measurement points in Fig. 6.

### Animal tissue

#### Top layer thickness

**Figure 7:**
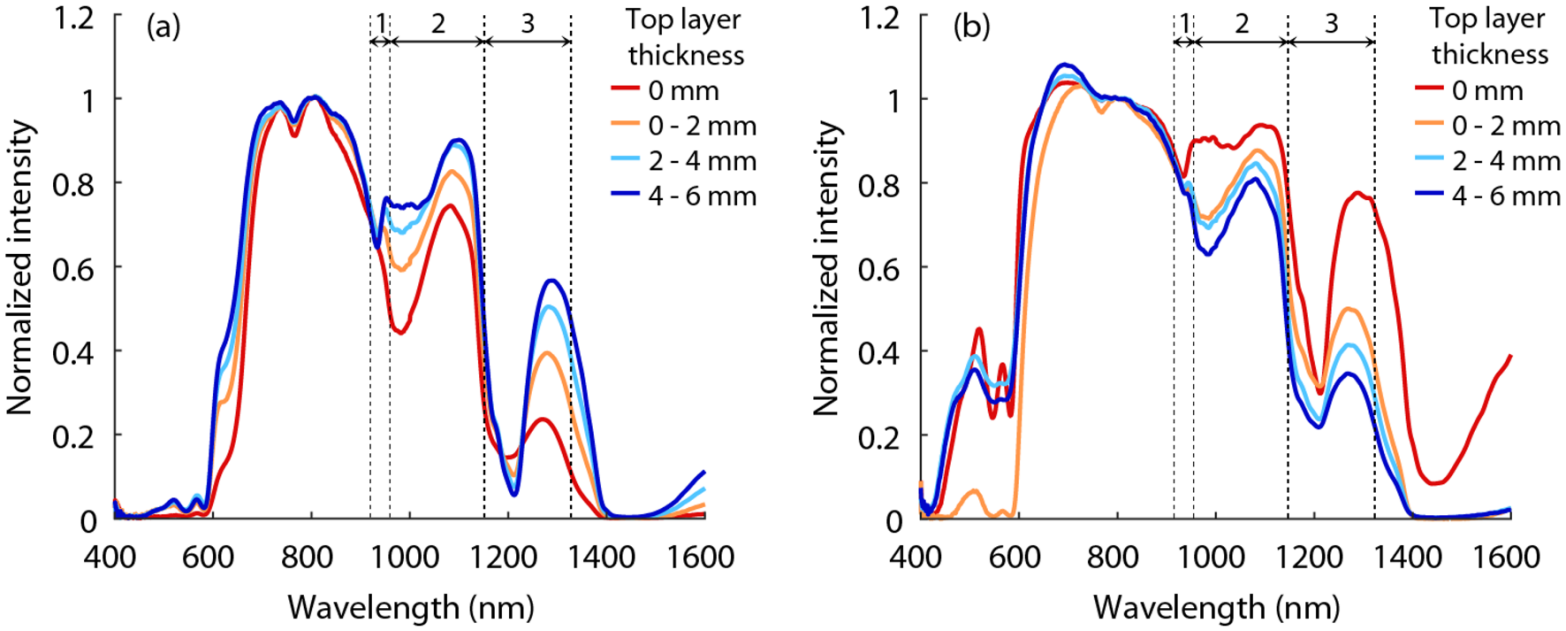
Mean diffuse reflectance spectra corresponding to different top layer thicknesses of fat on top of muscle tissue (**a**) and muscle on top of fat tissue (**b**), for a fiber distance of 6 mm. In the peak-based feature extraction method, the peak height was determined for the three outlined regions.

The average diffuse reflectance spectra for different top layer thicknesses are shown in Fig. 7. Again a clear relation between the top layer thickness as measured by the US and the measured spectra can be seen. The top layer thickness was predicted by performing regression analyses using the extracted DRS features from two fiber distances (2 and 6 mm) combined. The mean prediction errors and standard deviations are shown in Table 1. The exponential GPR model performs better than the linear SVM model. For a top layer of muscle tissue and the peak-based DRS features, significant differences were found between the results of the two regression models (p < 0.0001 and p < 0.001). Using the peak-based DRS features and the GPR model, mean top layer thickness prediction errors of 0.58 ± 0.55 mm and 0.43 ± 0.42 mm were found for fat on muscle tissue and muscle on fat tissue, respectively. To evaluate the added value of US features in the prediction of the top layer thickness, additional analyses were performed using the DRS features combined with US features. By combining the peak-based DRS features with the US features, mean top layer thickness prediction errors of 0.61 ± 0.51 mm and 0.35 ± 0.33 mm were found for fat on muscle tissue and muscle on fat tissue, respectively. Although the prediction errors are slightly lower in the case of a top layer of muscle tissue, no significant difference was found between the combined features and only DRS features for calculating the top layer thickness. On the other hand, using only the US features resulted in higher prediction errors of 1.23 ± 0.96 mm and 0.53 ± 0.60 mm for a top layer of fat and muscle tissue, respectively.

**Table 1:** Top layer thickness prediction errors, for both tissue types and feature extraction methods.

Feature set	Fat on muscle		Muscle on fat	
	Linear SVM	Exponential GPR	Linear SVM	Exponential GPR
DRS peaks	0.68 ± 0.56 mm	0.58 ± 0.55 mm	0.64 ± 0.55 mm*	0.43 ± 0.42 mm*
DRS wavelet	0.65 ± 0.56 mm	0.67 ± 0.60 mm	0.51 ± 0.39 mm	0.45 ± 0.33 mm
DRS peaks + US	0.70 ± 0.56 mm	0.61 ± 0.51 mm	0.60 ± 0.93 mm**	0.35 ± 0.33 mm**
DRS wavelet + US	0.66 ± 0.55 mm	0.66 ± 0.55 mm	0.48 ± 0.39 mm	0.39 ± 0.34 mm
US	1.62 ± 1.07 mm	1.23 ± 0.96 mm	0.75 ± 0.77 mm	0.53 ± 0.60 mm

*p < 0.0001, **p < 0.001.

**Figure 8:**
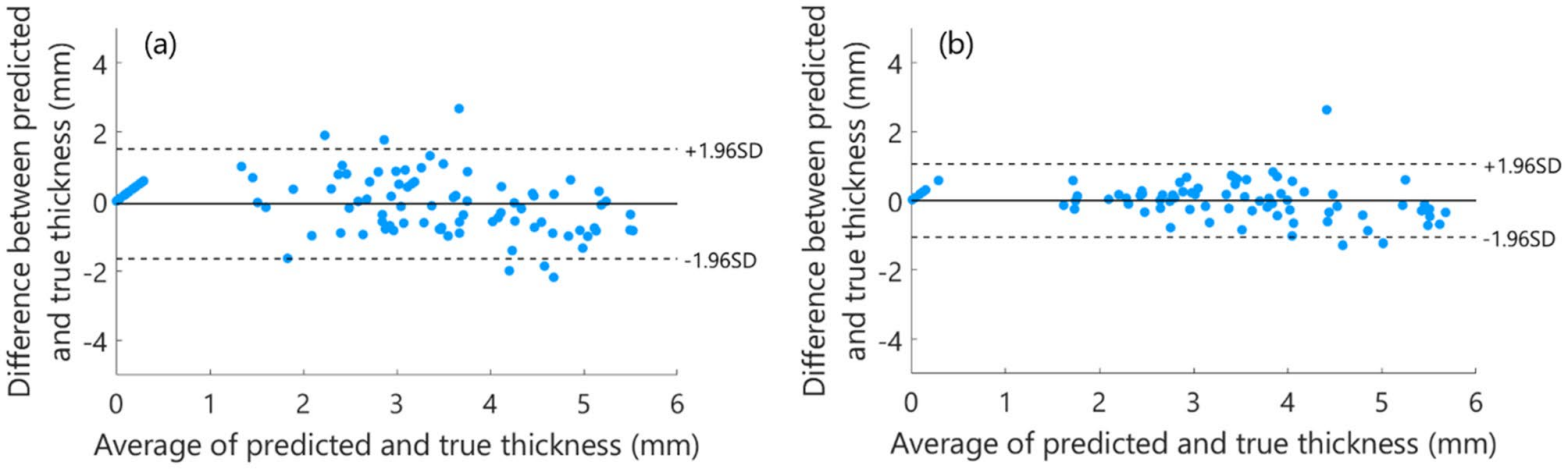
Bland–Altman plots of the agreement between reference values (ground truth) and predicted top layers thicknesses, using the peak-based DRS features and the GPR model, for **(a)** fat on top of muscle tissue and **(b)** muscle on top of fat tissue. The black line shows the mean difference between the predicted and true layer thickness, the dashed lines show the 95% confidence interval for the agreement (± 1.96SD).

The agreement between the predicted top layers thickness and the reference thickness can be observed in the Bland–Altman plots depicted in Fig. 8. Each plot shows the difference between the predicted and true thickness values against the average of them and permits a visual assessment of the distribution of errors. From the observation of these plots, it is worth mentioning that the results of our method are similar to reference values and do not show a substantial bias, as the mean of differences between the thickness values is close to 0. The 95% limits of agreement for fat on top of muscle tissue are slightly higher than for muscle on top of fat tissue.

#### Tissue classification

**Figure 9:**
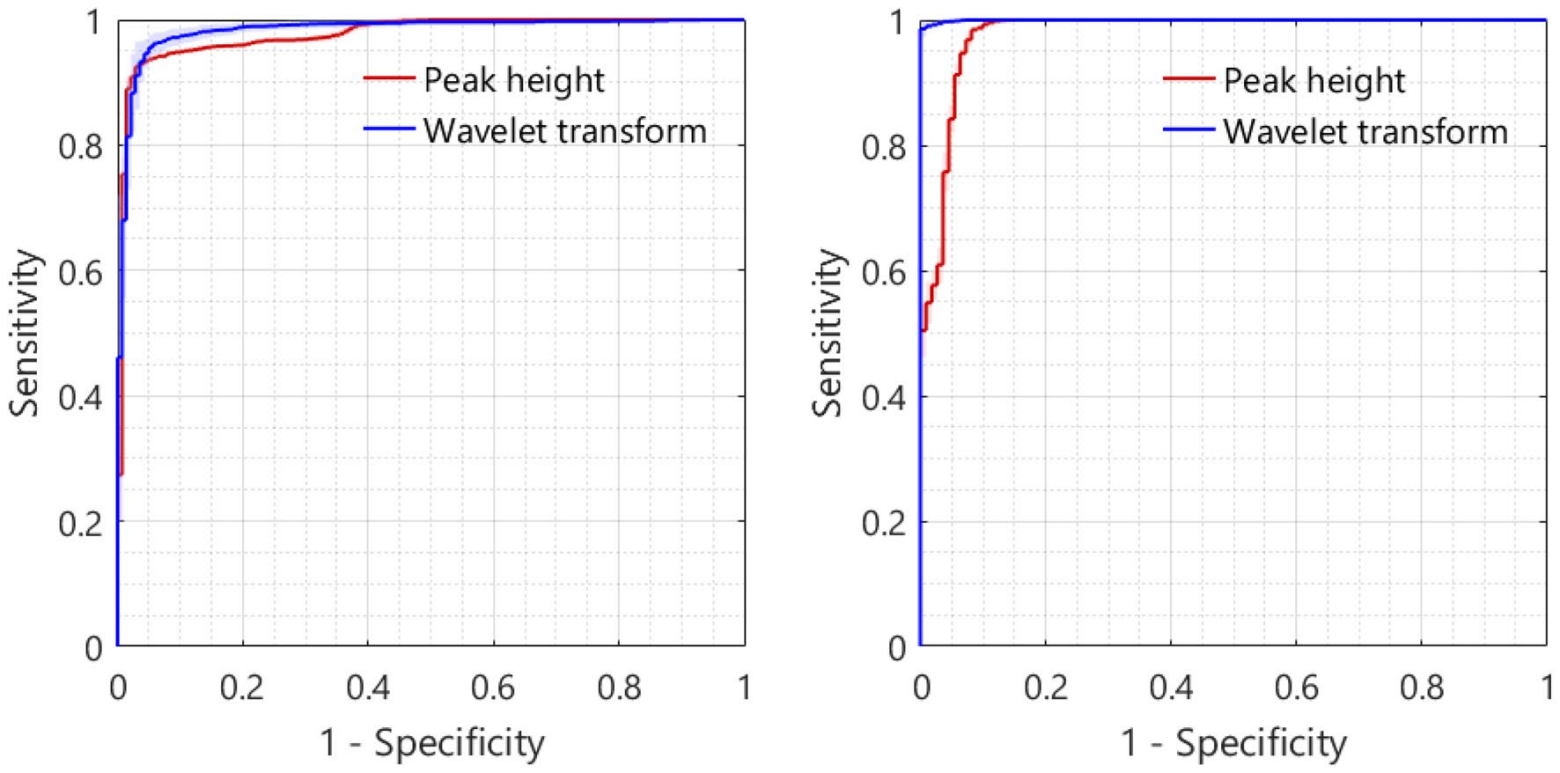
ROC curves of the tissue type classification for the first (left image) and second (right image) tissue layer, using the two feature extraction methods. The curves are averaged over the 20 iterations, the shaded area represents the standard deviation of the corresponding curve.

In Table 2, the mean classification accuracy, MCC, AUC, sensitivity, and specificity are shown for both layers and feature extraction methods. The classification accuracies for respectively the first layer and the second layer were 0.95 and 0.95 for the peak heights method and 0.95 and 0.99 for the wavelet method. The corresponding MCC values are 0.90, 0.90, 0.90, and 0.98. The ROC curves of the tissue classification are shown in Fig. 9.

**Table 2:** Tissue classification scores for both the first and second layers, using the two different feature extraction methods.

Performance metrics	First tissue layer		Second tissue layer	
	Peak heights	Wavelet transform	Peak heights	Wavelet transform
Accuracy	0.95	0.95	0.95	0.99
AUC	0.98	0.98	0.98	1.00
MCC^a^	0.90	0.90	0.90	0.98
Sensitivity^a^	0.92	0.94	0.98	0.98
Specificity^a^	0.97	0.96	0.92	1.00

^a^Fat was considered as the positive class.

## Discussion

In this study, the feasibility of predicting the top layer thickness and classifying the layers in two-layered phantom and animal tissue using diffuse reflectance spectroscopy was assessed. Data from two different source-detector fiber distances were combined to retrieve information from both superficial and deeper sampling depths. First, the prediction of layer thicknesses was examined in a controlled phantom study. Subsequently, the possibility of also classifying the tissue types of the different layers was examined using two-layered animal tissue. We have shown that there is a clear correlation between the top layer thickness and the spectral response at the patent blue absorption region, based on the phantom study (Fig. 5). The same principle is reflected in Fig. 7, which shows a similar experiment on animal tissue. Around 935 nm, 985 nm, and 1200 nm, clear differences could be seen between the spectra of different top layer thicknesses. For a top layer of fat, the intensity dip around 935 nm and 1200 nm becomes larger and sharper for larger top layer thicknesses, while the intensity dip around 985 nm becomes smaller. For a top layer of muscle, exactly the opposite applies. This can be explained by the fact that for a smaller top layer thickness, a larger part of the light travels through the second layer and the tissue type of the second layer becomes increasingly visible in the DRS spectrum.

In the first phase of this study, the top layer thickness of the artificial phantom could be predicted using a regression analysis with an accuracy of 0.69 ± 0.04 mm. Figure 6 also showed that this difference between the estimated thickness and the actual thickness of the first layer is small for all cases. These results indicated that it is possible to extract structural information from DRS spectra in a controlled two-layered phantom with perfectly homogeneous layers and sharp boundaries.

In the second phase, the top layer thickness of two-layered animal tissue structures could be predicted with accuracies of 0.58 ± 0.55 mm and 0.43 ± 0.42 mm using the peak-based DRS features for a top layer of fat and muscle tissue, respectively. The mean error is comparable to the value we found for the artificial phantom, while the standard deviation is larger in the case of the more realistic animal tissue. This difference in standard deviation could be caused by discrepancies between layer thickness measurements on CT and US images. Using the tissue-mimicking phantom, we compared the layer thickness measured with CT to the thickness measured with the US. A mean difference of 0.54 mm was observed. For the animal tissue phase, US was used as ground truth. Although the ultrasound probe was handled manually by the same operator during all measurements, small variations in measured thicknesses may occur due to differences in US probe pressure. Nevertheless, the results obtained using this proof of concept with animal tissue are promising since the errors are in the same order of magnitude as the US resolution (~ 0.5 mm) and well within the resection margin of a few millimeters that will ultimately be used during oncological surgery. Future research will have to show whether the accuracies remain comparable with the results of the current study when using the same framework on ex vivo tissue.

In addition, the added value of ultrasound imaging in estimating the top layer thickness was investigated. The combination of DRS and US features did not significantly improve the top layer thickness prediction accuracy compared to using DRS features only. The fact that the prediction accuracy using only DRS features was already in the same order of magnitude as the US resolution could explain why the addition of US features did not result in a significantly large improvement. In this study, we were dealing with controlled and ideal situations. In the future, we will have to deal with more complex situations. Using a multimodality approach, it may be possible to extract more information regarding the measured tissue, leading to valuable results.

The prediction error for the animal tissue with a top layer of muscle is smaller than for a top layer of fat, and this difference becomes more apparent when the US features are added. This might be due to the sharper boundary between the first and second tissue layers in the US images with a top layer of muscle. In addition, the US features are sensitive to structure and intensity differences within a layer, which is more common in fat tissue (see Fig. 4a). DRS is probably less sensitive to this, making this technique more reliable and robust. Table 1 clearly shows the difference in prediction error between fat and muscle tissue, when only the US features are used. This is not the case when using DRS features.

When comparing the results of the two regression models (Table 1), it can be seen that the Gaussian process regression model outperforms the simpler linear SVM model. In the case of the peak-based DRS features and a top layer of muscle tissue, the differences are significant (p < 0.0001 and p < 0.001). When looking at the two DRS feature extraction methods, the peak-based method performs slightly better than the wavelet-based method, except in the case of muscle on top of fat tissue and the use of the linear SVM model. This difference disappeared when using the more sophisticated GPR model. The curse of dimensionality also plays a role when comparing these two feature extraction methods. This is the phenomenon that increasing the dimensionality (number of features) of the model, without increasing the number of training samples, results in a decrease in classifier performance. On the one hand, the wavelet-based method takes information from the entire DRS spectrum into account, while the peak-based method focuses on only three specific areas of the spectrum. On the other hand, the wavelet-based method extracts 25 features per spectrum, while the peak-based method only extracts three. Machine learning models with more features are more prone to overfitting the data, which may decrease the prediction accuracy during testing. This might be the reason why the peak-based features, with less input information, still resulted in slightly better prediction accuracy.

In the first phase of this study, we showed that it was possible to predict the thickness of layers up to 14 mm, using source-detector fiber distances of 2 and 6 mm. This could be due to the low scattering and absorption of the phantom. In tissue, the sampling depth of DRS is expected to be roughly equal to the maximum fiber distance of 6 mm^49^. That is why in the second phase of this study, layer thicknesses up to 6 mm were included. Furthermore, it is not clinically relevant to measure much deeper than 6 mm from the resection surface.

In addition to determining layer thicknesses, the second aim of this paper was to classify the different tissue types of two-layered tissue. Good classification accuracies were achieved for both the first and second tissue layers of the animal tissue. The tissue type of the first layer was classified with an accuracy of 0.95 and an MCC of 0.90 for both feature extraction methods. The tissue type of the second layer was classified with an accuracy of 0.95 and 0.99 and an MCC of 0.90 and 0.98, for the peak heights method and wavelet transform method respectively. Both methods performed equally for the first layer, while the wavelet transform method performed better for the second layer. The most striking difference lies in the specificity of 100% for the wavelet transform method, which positively influenced the accuracy, AUC, and MCC. The main difference between the two feature extraction methods is that the peak-based method focuses on certain regions in the spectra, while the wavelet transform uses the entire spectrum. This may explain why the wavelet transform method performs better in certain situations.

In the current study, we only evaluated the added value of US features for the top layer thickness prediction and not for the tissue type classification. However, it would be interesting to investigate this further in future work. Different tissue types show different intensities and textural characteristics on US images. Extracting these types of US features, next to the DRS features, may improve the tissue type classification accuracy of more complex situations in the future.

The peak-based DRS features were extracted from fat and water absorption areas in the spectrum; 935 nm, 985 nm, and 1200 nm. For tissue discrimination in clinical practice, these areas will be important as well. Previous research showed that differentiation between tissue with high fat and high water content is important in multiple oncological domains. De Boer et al. showed, for example, that the fat and water content differs between benign and malignant tissue in breast cancer surgery and that the optical parameter related to the absorption of fat and water (F/W-ratio) provided the best discrimination between healthy and tumor tissue^21^. In colorectal cancer surgery, the tumor is assessed from outside the lumen and should be distinguished from fat and healthy colorectal wall, in contrast to colonoscopy where the tumor is assessed from the inside of the colorectal lumen. Baltussen et al. examined several options to optimize the classification of healthy colorectal wall versus tumor tissue and showed that tissue optical features, in which tissue constituents such as water and fat content were determined per spectrum using a fit algorithm, improved classification results the most^53^. For more complex situations, such as discriminating tumor versus muscle tissue, other discriminative features using more advanced feature extraction techniques can be used. Baltussen et al. showed that it is possible to discriminate healthy fat and colorectal muscle tissue from tumor tissue, in both ex vivo and in vivo settings, using specific spectral bands that were selected by k-means clustering^32^. In the current study, we showed this by using the wavelet transform feature extraction method, which is extracting meaningful features over the entire wavelength range.

Previous studies in literature focused primarily on identifying the tissue directly at the surface, assuming single-layer homogeneous tissue to be present. In this study, however, not only the top layer was examined, but we went a step further and also examined the layer below it up to 10 mm in depth. To do this, we combined DRS features from two different source-detector fiber distances (2 and 6 mm) to retrieve information from both superficial and deeper sampling depths. The combination of tissue type and thickness of the first two layers is incredibly valuable information and can be used to provide intra-operative guidance for surgeons in order to achieve accurate resection margins and preserving healthy tissue as much as possible. In addition, Baltussen et al. examined the influence of tumor depth from the measurement surface on the classification accuracy when assuming one homogeneous tissue volume^33^. They showed that the classification accuracy drops from a tumor depth of 1.5 mm, using a fiber distance of 2 mm. The two-layer approach and the combination of multiple fiber distances proposed in this study may be a solution for this issue.

In this study, two different experiments using a phantom and animal tissue samples were performed to examine the algorithm performance. However, it still concerned simple set-ups to investigate the feasibility of our proposed two-layer approach in a controlled situation. Animal fat and muscle tissue samples were examined, which may optically differ only slightly from human fat and muscle tissue. In addition, in clinical practice, more tissue types will be encountered, the tissue layers will be more heterogeneous and the boundaries on the US image might be less clear. Ultimately, the challenge will be to distinguish tumor tissue from multiple types of healthy tissue. The next step would, therefore, be to test the performance of our two-layer approach and developed algorithms in a more realistic situation with surgically excised tissue (ex vivo study) before our method can be used in in vivo clinical practice. When retraining the developed models for this new situation and dataset, the models will also take into account any differences in chromophore concentration within one tissue type between different patients during the layer thickness prediction. The proof of concept presented in this study, in particular the framework for layer thickness prediction and layer classification, can be extended to other tissue types and other medical and non-medical applications.

## Conclusion

We introduced a new pipeline for predicting the top layer thickness and classifying the layers in two-layered phantom and animal tissue using diffuse reflectance spectroscopy. The top layer thickness was predicted with an accuracy of up to 0.35 ± 0.33 mm and the tissue type of the first and second layer could be classified with an accuracy of 0.95 and 0.99, respectively. These results show the potential of the proposed technique in revealing more complex tissue architectures and extract information from larger sampling depths. An ex vivo study in which more tissue types will be encountered is the next step towards reaching the ultimate goal of evaluating resection margins up to multiple millimeters in depth during oncological surgery.

## Data Availability

The datasets generated during and/or analyzed during the current study are available from the corresponding author on reasonable request.
